# Acute Transverse Myelitis in Children, Literature Review

**Published:** 2018

**Authors:** Azita TAVASOLI, Aidin TABRIZI

**Affiliations:** 1 Department of Pediatric Neurology, Iran University of Medical Sciences, Tehran, Iran.; 2 Pediatric Neurology Research Center, Shahid Beheshti University of Medical Siences, Tehran, Iran.

**Keywords:** Acute transverse myelitis, Acquired demyelinating syndromes, Childhood transverse myelitis, Myelitis

## Abstract

**Objective:**

Acute transverse myelitis (ATM) is a rare inflammatory demyelinating disorder characterized by relatively acute onset of motor, sensory, and autonomic dysfunction. Children comprise 20% of total cases of ATM. In this review, we described the current literature on childhood ATM, focusing on the epidemiology, pathogenesis, clinical presentation, approach to diagnosis, differential diagnosis, treatment and outcome in the pediatric population.

**Materials &Methods:**

We searched the related articles in electronic databases such as Scopus, EMBASE, Google Scholar, and PubMed. All study designs were included and the essential key words for searching were myelitis, acute transverse myelitis, childhood transverse myelitis, and acquired demyelinating syndromes.

**Results:**

The related data focusing on the epidemiology, pathogenesis, clinical presentation, diagnostic approach and differential diagnosis, treatment and outcome of pediatric ATM were gathered and described.

**Conclusion:**

ATM is a heterogeneous disorder in children with a broad spectrum of clinical presentation, etiology, and outcome. It may be the first presentation of relapsing acquired demyelinating syndromes and also must be distinguished from compressive and noninflamatory myelopathies. Correct diagnosis is crucial for treatment and prognosis.

## Introduction

Childhood acute transverse myelitis (ATM), an inflammatory involvement of spinal cord, is a rare demyelinating and immune-mediated disorder of central nervous system (CNS) (1). It is potentially a devastating disorder with variable outcomes that characterized by relatively acute onset of motor, sensory, and autonomic dysfunction (2). ATM may be the harbinger presentation of relapsing acquired demyelinating syndromes (ADS) such as multiple sclerosis (MS), acute disseminated encephalomyelitis (ADEM), and neuromyelitis optica (NMO). It can occur, however, as an isolated condition, usually after an infectious disorder. It is important to distinguishing ATM from compressive and noninflammatory myelopathies. Diagnostic evaluation, prognosis, recurrence risk, and treatment plans are different among these entities.

In this review, we described the current literature on childhood ATM, focusing on the epidemiology, pathogenesis, clinical presentation, approach to diagnosis, differential diagnosis, treatment and outcome in the pediatric population. 

## Materials &Methods

We searched the related articles in electronic databases such as Scopus, EMBASE, Google Scholar, and PubMed. All study designs were included and the essential key words for searching were myelitis, acute transverse myelitis, childhood transverse myelitis, acquired demyelinating syndromes. The related data focusing on the epidemiology, pathogenesis, clinical presentation, diagnostic approach and differential diagnosis, treatment and outcome of pediatric ATM were gathered and described.

## Results

**Epidemiology**

The incidence of pediatric ATM is 1.7-2 per million children yearly (3). Male to female ratio is 1.1–1.6:1 (1), but a female predominance is reported in teenager cases associated with MS or NMO (4–6). ATM occurs in adults more common. Children comprise 20% of total cases of ATM (7). ATM encompasses one-fifth of pediatric cases that present with a first ADS(8). Age distribution of pediatric ATM is bimodal primarily affected children under 5 and older than 10 yr of age (9,10) . There is no ethnic predisposition (1). A preceding infection or vaccination is reported in up to 66% and 28% of children with ATM respectively, during 30 d before the onset of the disease (7,9,11) . Mild spinal trauma or allergic shot may be preceding risk factors (12).

**Clinical features:**

The first presentation of ATM in children is usually back pain. Rapidly progressive motor deficits develop in the lower extremities. Initially, flaccid paresis and decreased deep tendon reflexes (DTRs) are often detected, but subsequently, this condition evolves to a state of increased tone and increased DTRs below the level of lesion during subsequent days up to 12 wk (12). Upper extremities also may be involved in the spinal cord lesion is in the thoracic region (12). Sensory deficits such as pain, burning paresthesia, hyperesthesia and numbness and sphincter dysfunction are also present (9,13). Urinary retention resulting in catheterization is seen in the most of the children (7,9). Autonomic dysfunction is common including variations in body temperature and instability in respiratory rate as well as heart rate and rhythm; also, urinary symptoms such as urgency, incontinency, and difficulty in voiding have been reported. Patients can experience intestinal dysfunction as constipation or incontinency(12,14). Priapism and visual loss have also been reported (12). Urinary retention may be the first presentation of myelitis (15). It is seen in 95% of the patients during the acute phase and its reason is disruption of the signal between the micturition center in the pons and the sacral level (12). Sensory level is detectable in the majority of patients mostly in the thoracic region (12). The border of sensory level establishes the level of spinal cord involvement that is an essential factor in determining the prognosis of the disease (16). In up to 40% of children, a clear sensory level may not be assessed (9,10,17). Neurologic symptoms progress during next 2-4 d and reach a nadir within 5-6 d of onset (6,9,18). The length of the plateau phase of symptoms is 1 wk (range, 1-40 d) (12).

Initial recovery occurs nearly 9 d after the onset of the disease (range, 2-50 d) (12). The first symptoms to resolve after starting treatment are pain and then motor deficits. Sensory deficits and urinary dysfunction may need longer time to resolve(9). The time to walking is the least predictable (ranges 2 d- 1 yr) (12). 

Subtypes of ATM are described based on the clinical severity as well as the nature and extent of spinal cord involvement in imaging. Acute complete transverse myelitis (ACTM) includes symmetric motor, sensory and autonomic dysfunction on both sides below the level of the lesion concurrent with MRI lesions in one to two vertebral segments. In acute partial transverse myelitis (APTM), clinical presentation can be mild or asymmetric with involvement of one to two vertebral segments in MRI. Longitudinally extensive transverse myelitis (LETM) describes complete or incomplete dysfunction of the spinal cord with a lesion extending over three or more vertebrae (9,10,17). LETM occurs in 66%-85% of pediatric ATM (2,5) and could be associated with NMO and ADEM- associated TM (7). Otherwise, MS is frequently associated with segmental TM involving fewer than three vertebral segments (12). An enhancing signal abnormality can be usually seen over one or more vertebrae in spinal cord MRI (9). Absence of gadolinium-enhancing of the lesion does not rule out ATM (2,5,7). Essentially, the lesions are centrally located within gray matter and can be discrete or contagious (5). The most lesions are cervical (64%-76%) and cervicothoracic (5,7,18). Generally, brain MRI is normal in idiopathic ATM, although asymptomatic lesions are seen in more than 40% of the patients and predict a higher risk of developing NMO or MS (2). Therefore, brain MRI is necessary for the evaluating TM patients (10). MS often (66%-88%) occurs in children with APTM associated with cerebral lesion in MRI (10,17). 

Cerebrospinal fluid (CSF) findings are lymphocytosis (usually less than 100/mm3) and increased protein level (usually 100-120 mg/dl). In 20%-50% of children with definite ATM, CSF analysis shows normal protein levels and white blood cells count (5,7,9,11). In isolated ATM, oligoclonal bands (OCBs) in CSF usually are not detected. However, OCBs are elevated in one-third of LETM patients (12). If OCBs are detected in CSF, an increased risk of subsequent MS is expected; subsequent MS is expected (19,20).

The most cases of ATM develop as an isolated post infectious disease (idiopathic ATM), but ATM can occur associated with other neuroinflammatory disorders including NMO, MS, and ADEM (secondary or disease-associated ATM). ATM can be associated with some other CNS disorders consisting of infections due to some pathogens such as Cytomegalovirus, Herpes viruses, Enteroviruses, Epstein-Barr virus, influenza virus, hepatitis A,B and C viruses, West Nile virus, HTLV1, mycoplasma, Varicella-Zoster virus (21), Zica virus (22), tuberculosis, neurosarcoidosis; and paraneoplastic syndromes. A few systemic inflammatory diseases also can be associated with ATM including systemic lupus erythematosus (SLE), mixed connective tissue disease (MCTD), primary Antiphospholipid antibody syndrome, Sjogren syndrome (SS) and Behcet disease (23–25).

**Pathogenesis**

Histologic findings are different in idiopathic and disease-associated ATM, but inflammation and neural loss are seen in both (26). Monocyte and lymphocyte infiltrations in the lesion and axonal degeneration are reported and both gray and white matters are involved (27). In fact, ATM is a combined inflammatory disease that involves multiple components of CNS including neurons, axons, oligodendrocytes, and myelin rather than a pure demyelinating disease (26). Histopathologic studies in adults show intralesional infiltration of monocytes and CD4+ and CD8+ T lymphocytes associated with astrocyte and microglia activation (28). Necrosis and cavitation may occur especially in NMO, resulting in severe disability (29). There have been described two potential mechanisms of autoimmunity in ATM including molecular mimicry and superantigen effect (26). A pediatric study showed significant increase in the Interleukin-6 (IL-6) levels in the CSF of the children with ATM (30). IL-6 plays role in cellular injury of the spinal cord. An association between increased IL-6 levels and disability has been shown (31). Studies showed a decreased IL-6 response to monoclonal antibody trial in early phase of ATM with appropriate outcome in NMO and ATM patients (32). Various autoantibodies are implicated in ADS by crossing the blood-brain barrier such as aquaporin-4 and myelin oligodendrocyte glycoprotein (MOG) antibodies in NMO and childhood ADS, respectively. The latter may be predicted a non-MS course (33). Some autoantibodies also may directly damage neurons that expose antigens which cross-react with antibodies against infectious organisms (34).

**Diagnosis and evaluation**

The diagnosis of TM is proposed when the patient present with signs and symptoms of bilateral sensory, motor and autonomic dysfunction localized to one or more spinal segments without evidence of a cord compression. Therefore, the following criteria are necessary for the diagnosis of ATM:

1- Exclusion of compressive lesions, and

2- Confirmation of spinal cord inflammation as detected by the following:

2-1) the gadolinium enhancing lesion in MRI, or 

2-2) CSF evidence of either pleocytosis or elevated immunoglobulin type G (IgG) index

However, the lack of the inflammation markers does not exclude ATM (23). Developing symptoms of myelopathy considered as an emergency condition since severe squealed can occur if the disorder is not diagnosed and treated promptly (1). Emergent spinal MRI is required to exclude a compressive lesion. After that, determination of inflammatory or noninflammatory myelopathy is necessary according to above-mentioned markers. If inflammation is present and TM is suspected, then some investigations are recommended (35). 

Brain MRI with and without contrast for detecting MS or ADEM; CSF analysis for cell count, protein, glucose, OCBs, IgG index, and cytology; serum NMO IgG antibodies (anti-aquaporin-4 IgG); serum B12; methylmalonic acid; human immunodeficiency antibodies; thyroid function test; antiphospholipid antibodies, antinuclear antibodies, rheumatoid factor and anti-dsDNA (35). In more than 40% of children, asymptomatic brain MRI lesions are seen and may be a risk factor for developing MS or NMO (2). Ophthalmologic consultation for detecting comorbid optic neuritis is recommended for all patients (12). Some children with encephalopathy and young children may not have complaints of vision impairments (12). Additionally, neurophysiological findings of subclinical optic neuritis may exist (12). 

**Differential diagnosis**

Three categories in the differential diagnosis of idiopathic ATM are as follows: 

1. Other forms of myelopathy such as compressive or noninflammatory including epidural hematoma, intervertebral disk herniation, vertebral body fracture, ischemic myelopathy due to arterial compromise or venous hypertension (36). Spinal cord tumors occasionally present with subacute pain and myelopathic symptoms. Extramedullary tumors consist of nerve sheet tumors, meningioma, and metastasis of medulloblastoma. Intramedullary tumors include astrocytoma and ependymoma (1). Spine tuberculosis is another cause of compressive myelopathy. Other noninflammatory causes are vitamins deficiency including B12, D, and E along with copper deficiency (35).

2. Secondary ATM including cases due to identified causes such as infectious myelitis, a rheumatological disease (e.g. SLE and SS), paraneoplastic syndromes, demyelinating CNS disease (e.g. ADEM, MS, and NMO). Enteroviruses have recently been reported to be mediated in acute flaccid myelitis due to direct invasion to motor neurons of spinal cord (37). Clear diagnosis of disease-associated TM by clinical and paraclinical features is important since the prognosis, treatment and recurrence risks are different (1). Patients with NMO typically have LETM (38).

3. Non myelopathic disorders can mimic ATM essentially Guillain-Barre syndrome (GBS). Clinical features of GBS including acute weakness and progressive motor and sensory dysfunctions resemble to ATM (39). However, some clinical and paraclinical factors help to discriminate them. Autonomic involvement in ATM present with intestinal or urinary urgency or retention, rather patients with GBS have cardiovascular instability. Sensory level is characteristic in ATM, while is never detected in GBS. IgG index in CSF and distinct spinal cord lesion in MRI are two findings in ATM but not detected in GBS patients. Finally, in GBS, conduction block or delayed conduction of peripheral nerves may be seen in electro diagnostic studies, but these studies are usually normal in ATM patients (1). 

Regarding the onset time of the symptoms of spinal cord syndrome, differential diagnosis are classified as follows: symptoms with acute or hyperacute onset should prompt consideration of spinal infarct, hemorrhage or disk herniation. Slowly progressive onset of the symptoms is in the favor of compressive myelopathy such as tumor, nutritional deficits, toxin exposure and hereditary disorders such as hereditary spastic paraplegia. Subacute presentation is in the favor of demyelination (TM or NMO), infections, and vasculitis (SLE)

**Treatment**

US Food and Drug Administration FDA approved no treatments for ATM since lack of controlled clinical trials (1). Therapies are according to the data from open-label and retrospective studies. In specific conditions, certain medications have better therapeutic results (40), such as cyclophosphamide therapy in SLE patients with ATM and plasmapheresis (PLEX) in patients with NMO. Standard first-line therapy in idiopathic ATM is intravenous high dose corticosteroids that are prescribed as 30 mg/kg/d (maximum 1 gr/d) of methylprednisolone for 3-7 d (1,12,41). Outcome of the patients with mimics of ATM does not worsen by corticosteroids. Thus, the benefit of starting earlier therapy is preferable (1). The patients treated with corticosteroids have better short and long-term outcome (41,42) and the time of disability is shorter (43). Intravenous methylprednisolone should be followed by an oral steroid starting at 1 mg/kg/d and tapering over 3-4 wk (12,44). Plasmapheresis should be considered if clinical response does not begin or symptoms worsen during 24-48 h after starting steroids (12). Some centers use PLEX concurrent with steroids when significant motor or respiratory dysfunctions are present (45,46). Waiting until completing steroids therapy is not necessary for patients with serious deficits. Though, according to American Academy of Neurology guidelines published in 2011, benefits of PLEX is recognized in adult ATM patients (12). The preferred protocol is 5-7 treatments, each exchanging 1.1-1.5 plasma volumes and every other day for 10 d (47). Intravenous immunoglobulin 2 gr/kg divided over 2-5 d is often used in fulminant cases, though limited evidence exist for its benefits (12,17). Cyclophosphamide 500-750 mg/m^2^ have been used in patients with SLE for improved outcome (12). Chronic immunomodulatory treatment by drugs such as azathioprine, methotrexate, or mycophenolate should be considered in patients with recurrent disease (34). 

In patients with the diagnosis of MS or NMO also, typical immunomodulatory drugs should be initiated after treatment of acute period (48). Consultation with physical medicine and rehabilitation specialists is necessary for beginning rehabilitation soon as soon possible (12). Bladder & Bowel continence programs should be initiated. In this regard, anticholinergic drugs such as oxybutynin or tolterodine may be helpful. In some patients, drugs such as gabapentin, carbamazepine, phenytoin, amitriptyline or baclofen may be needed to relieve pain (12). 

**Prognosis and outcome**

Outcome of ATM in children is better than adults, as almost 50% of children obtain recovery after 2 yr (7,49). In general, outcomes are very different from complete recovery without sequelae to entire paralysis and even death (12). 33%-50% of the patients show complete recovery and 10%-20% of cases have poor outcome (12,50). Infants have the worst outcome (43). More frequency of LETM in infants and inability of immature brain to recover from injury in them are two reasons (12). Recovery phase can continue up to several years (12,51). Death is usually due to a high cervical cord injury and respiratory insufficiency (7,41). Sensory deficits and urinary dysfunction are the most common sequelae (15%-50%) (2). Nearly 10%-20% of patients never obtain mobility or urinary function (1). Poorer outcomes have been associated with: younger age at the onset of the disease, rapid onset of symptoms, shorter time (less than 24 h) to maximal deficit, complete paraplegia, need to assisted ventilation, longer time to treatment, absence of CSF pleocytosis, higher border of sensory level, T1 hypointensities in spinal cord MRI and longitudinal extent of the cord lesions (1,23,42,52). Prognosis is better in TM associated with ADEM (12). TM in most patients is monophasic, but 25%-33% of the patients with idiopathic TM and as high as 70% of patients with disease-associated TM develop recurrences (50). Factors that predict recurrence include: longitudinally extensive lesions in spinal cord, brain lesions on MRI, existence of one or more autoantibodies (ANA, ds DNA, phospholipid antibody, C-ANCA), underlying collagen vascular disease, OCBs in CSF, presence of NMO-IgG (anti-Aquaporin-4)antibody (27,50) and female sex (1). Patients with LETM have an increased risk for NMO.LETM, recurrent ATM, ATM with concurrent or rapidly sequential optic neuritis suggest NMO (12). In addition, OCBs can be detected in 30% of patients with NMO (12). Besides, patients with OCBs in CSF have an increased risk for evolving MS (12). According to a Canadian study, MS develops in 13% of children with ATM (8). In addition, patients with patchy lesions involving 1-3 segments in cord, partial myelitis, and brain lesions in MRI have a rate of 60%-90% progression to MS during 3-5 yr (53). A study has reported cognitive impairment in 10% of pediatric ATM patients. 

We suggested monitoring of this population for cognitive problems (54). Apart from initial outcome, ATM patients should be monitored longitudinally, either to clearing diagnosis or to provide rehabilitative interventions for motor deficits, urinary dysfunction, psychological and cognitive impairment (1).

**In Conclusion,** ATM is a heterogeneous disorder in children with a broad spectrum of clinical presentation, etiology, and outcome. It may be the first presentation of relapsing acquired demyelinating syndromes or occur as an isolated postinfectious condition. It also must be distinguished from compressive and noninflammatory myelopathies. Diagnostic evaluation, prognosis, recurrence risk, and treatment plans are different among these entities. Therefore, the correct diagnosis is crucial for treatment and prognosis.
